# Applied Swarm-based medicine: collecting decision trees for patterns of algorithms analysis

**DOI:** 10.1186/s12874-017-0400-y

**Published:** 2017-08-16

**Authors:** Cédric M. Panje, Markus Glatzer, Joscha von Rappard, Christian Rothermundt, Thomas Hundsberger, Valentin Zumstein, Ludwig Plasswilm, Paul Martin Putora

**Affiliations:** 1 Department of Radiation Oncology, Kantonsspital St. Gallen, Rorschacherstrasse 95, 9007 St. Gallen, Switzerland; 2 Department of Nephrology, Inselspital Bern, Bern, Switzerland; 3 Department of Medical Oncology, Kantonsspital St. Gallen, St. Gallen, Switzerland; 4 Department of Urology, Kantonsspital St. Gallen, St. Gallen, Switzerland

**Keywords:** Decision tree, Consensus, Consensus finding, Cancer, Radiotherapy, Swarm-based medicine

## Abstract

**Background:**

The objective consensus methodology has recently been applied in consensus finding in several studies on medical decision-making among clinical experts or guidelines. The main advantages of this method are an automated analysis and comparison of treatment algorithms of the participating centers which can be performed anonymously.

**Methods:**

Based on the experience from completed consensus analyses, the main steps for the successful implementation of the objective consensus methodology were identified and discussed among the main investigators.

**Results:**

The following steps for the successful collection and conversion of decision trees were identified and defined in detail: problem definition, population selection, draft input collection, tree conversion, criteria adaptation, problem re-evaluation, results distribution and refinement, tree finalisation, and analysis.

**Conclusion:**

This manuscript provides information on the main steps for successful collection of decision trees and summarizes important aspects at each point of the analysis.

## Background

Evidence-based medicine (EBM) is the conscientious, explicit, and judicious use of current best evidence in making decisions about the care of individual patients [1]. While randomized controlled trials are considered to be the highest level of evidence [2], they are not available for a significant proportion of clinical decisions [3, 4]. In the absence of evidence several compensatory strategies are being applied [5]. Due to technological advances, barriers in communication within the medical community have been massively reduced enabling swarm-like behavior of medical communities. Swarm-based medicine [6], as a form of collective intelligence, represents an additional source of information in decision making [6, 7].

In order to obtain information on medical decision making from a swarm, i.e. the medical community, medical information from individual medical care providers [8–10] or different guidelines [11] needs to be collected in a homogenous format for subsequent comparison and analysis.

Decision trees can be used to visualize complex treatment strategies and to compare treatment algorithms of different individual entities [12]. When these are constructed with the same rule-set and terminology, their comparison becomes possible [13]. By analysing recommendations from every party for every permutation of decision criteria, the majority decision for each permutation can be established. The advantage of such an approach is that the risk of bias through moderation or bias towards individual trees is eliminated, this has been termed the objective consensus methodology [14]. This approach has been involved in various settings in clinical oncology [15–18].

The aim of this manuscript is to describe the steps that are required to collect, refine and analyse decision trees to determine consensus and controversy among experts, guideline panels or healthcare institutions for the purpose of identifying trends in medical decision making within a specified clinical setting.

## Methods

The coordinating physicians from five finalized and two ongoing cancer clinical care projects investigating patterns of algorithms (treatment recommendations) were asked to summarize the steps that were required to establish an analysis of multiple decision trees using the objective consensus methodology [14]. The five concluded projects involved an analysis of patterns of care of radiotherapy and androgen deprivation therapy for prostate cancer in Switzerland [15], the multidisciplinary management of recurrent glioblastoma in Switzerland [17] and a review of systemic therapies for metastatic clear-cell renal cell cancer among international experts [16, 18]. One project is investigating recommendations of international guidelines for urolithiasis.

The ongoing projects investigate published guidelines on Pompe’s disease, a rare metabolic myopathy, and patterns of chemotherapy for metastatic pancreatic cancer, respectively.

The coordinators (CMP, TH, CR, JvR, VZ, MG) of the projects were asked to describe their projects in an individual list of steps performed (and problems encountered) during their respective projects. The steps within these unformatted lists were manually compared (by PMP) and steps with similar descriptions merged. All coordinators participated in an unstructured discussion on finding a common nomenclature and description for these steps. The coordinators provided complementary information and no conflicting experiences or recommendations were reported. This resulted in a descriptive procedure aimed at guiding investigators through this process, while pointing out caveats experienced by the authors during the concluded projects.

## Results

**Fig. 1:**
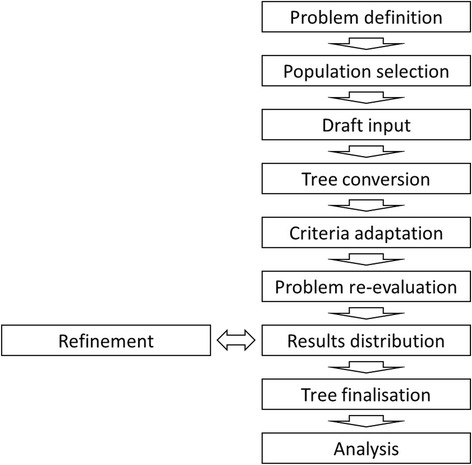
Workflow for patterns of algorithms analyses based on decision trees

Based on iterative discussions nine steps that applied to all seven projects were identified (Fig. 1). The following text will describe procedures within the individual steps as well as problems associated with them.

### Problem definition

The research question is defined and needs to be suitable for a decision tree analysis.

Implementing an objective consensus methodology is associated with a certain workload, which is only cost-efficient for problems which are complex enough to justify the effort. For a simple yes-no question based on 1 or 2 binary criteria a simple table would be a more efficient approach, classical surveys and accompanying discussions would be a more efficient solution where multiple simple questions are not directly linked to each other [10]. The other extreme would be represented by decisions that are so complex that it is not realistic to expect a clinical expert or guideline to even explicitly describe every possible scenario (such as a comparison of every treatment for every cancer for every type of patient), such a complexity may be prohibitive.

It can be expected that decisions, which mostly rely on individual factors such as patient preference, and other parameters which are difficult to objectify will not be adequately represented by decision trees.

To keep the scientific question on medical decision-making clearly understandable, it is favourable to focus on a specific medical condition to allow a detailed analysis of treatment algorithms. The problem investigated needs to be specific enough to be categorizable. It is pragmatic to avoid being too complex or extensive to keep the workload in answering the questions reasonable for the participants. For instance, the survey on primary external beam radiotherapy of prostate cancer [15] with and without androgen deprivation did not include a comparison with other treatment modalities such as surgery, brachytherapy or high intensity focused ultrasound (HIFU), to keep the scientific question focused and to allow for a detailed analysis of treatment parameters related to the main question.

### Population selection

A representative and unbiased group of participants is selected.

One of the challenging issues encountered during the mentioned projects included the fact that often a precise cut-off for the target population (the “swarm subset”) was difficult to define. When international experts are the target [16, 18], no unprejudiced cut-off for whom to include or exclude is possible. This was also critically pointed out by the reviewers during the submission process of these respective manuscripts. In the case of national or territorial projects this issue should not pose a problem, as in the survey on prostate cancer radiotherapy in Switzerland [15] where all centres in Switzerland were asked to participate. If an analysis of different guidelines is performed, population selection criteria and different health care systems must be defined and have to be addressed as well (i.e. age limits in UK). In an ongoing project analysed guidelines on the surgical management of urolithiasis, all guidelines were selected based on the member list of the international society of urology (SIU).

Ideally, group selection is explicitly defined, objective and reproducible; if this is not possible, it must be clearly stated that any conclusions from the analysis are biased by the selection of the representatives being analysed [16]. For instance, a study on treatment options for recurrent glioblastoma defined the inclusion criteria for the survey as a representative of a tumour board of a neuro-oncology centre with a scientific affiliation with the Swiss Group for clinical cancer research (SAKK) and the presence of an integrated neuro-surgery, radiation oncology and medical oncology unit [17]. A study on prostate cancer radiotherapy in Switzerland included all hospitals with an independent unit for external beam radiotherapy. For each centre, a board-certified radiation oncologist was contacted who had the competence to represent the current practice of his institution [15].

In a complex multimodality setting, the representatives of the different centers need to be able to apply various modalities. In case of the recurrent glioblastoma project in Switzerland, individuals from each center represented their respective multidisciplinary tumour boards. The individuals were required to discuss their institutional treatment recommendation with other specialists (e.g. if the information was provided by a radiation oncologist, the recommendation was approved by the neuro-surgeon and the medical oncologist). This is critical as otherwise a bias based on individual strategy within a center or bias based on one’s own specialisation is probable [19, 20].

### Draft input

The first unformatted input from the participants is collected.

Based on our experience, in most centers, medical treatments are not always explicitly defined in institutional standard operating procedures or other forms of explicit guidelines. And even if standard operating procedures exist explicitly, they may not cover every possible eventuality. Additionally, it cannot be expected that participants produce a complete and non-conflicting valid decision tree at their first attempt without previous experience within a similar project. For this reason, the first round of collected input can be automatically regarded as draft input. Furthermore, sufficient time and effort needs to be reserved to explain the concept of the objective consensus methodology to the participants.

It is advisable to collect the data anonymously and without the knowledge of the input from other centers to avoid any bias. The participants should be provided with the question alone without any examples or sample answers. This is important as any predefined answers may be suggestive and may influence the provided recommendation. For several treatment strategies, the general state of the patient is relevant, this may be represented by criteria such as fitness, age, comorbidities or performance status. It is important not to predefine these terms to be able to differentiate later whether e.g. age is an independent factor to fitness or performance status. Also, when inquiring what the treatment recommendation would be for patients over 70 years of age, the actually used cut-off value of 65 years in a specific case could be supressed.

**Fig. 2:**
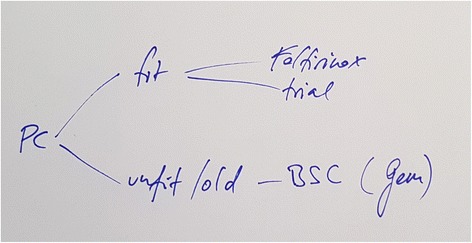
A sample draft decision tree, containing several imperfections**.** Text below the figure: (PC pancreatic cancer, BSC best supportive care, Gem gemcitabine)

The provided example (Fig. 2) shows a very simple and first rough decision tree for metastatic pancreatic cancer (PC). Despite its many flaws (age and fitness not clearly differentiated, it is not clear when “GEM” (gemcitabine) is really used instead of best supportive care and two recommendations for fit without differentiation are provided), this is a first important step for further discussion.

When guidelines are the target for analysis, at least two authors should extract the recommendations from the guidelines independently, followed by an appraisal where potential discrepancies are discussed and resolved.

### Tree conversion

Input from participants is converted into decision trees.

**Fig. 3:**
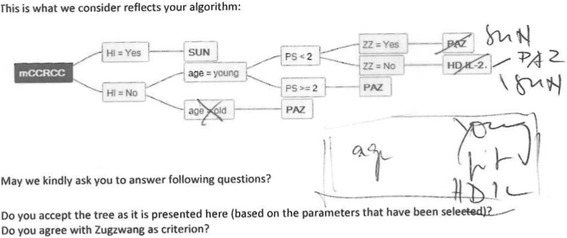
A sample of the first decision tree sent to the participating centre for validation and correction. Text below the figure: At the stage of refining the decision trees, any format can be used (in this example we used a decision tree in an email, which was discussed over phone, printed out and corrected with pencil). mCCRCC: metastatic clear cell renal cancer HI: hepatic insufficiency, SUN: sunitinib, PS: performance status, ZZ: Zugzwang, PAZ: pazopanib, HD IL-2: high-dose interleukin 2)

The first input may be provided in any format. Participating individuals should be burdened as little as possible with technical issues, as this may affect the return rate negatively; a rough incomplete paragraph on their strategy can be very helpful to start the interaction and to refine the details in an iterative approach. First versions of decision strategies were collected in various text-based and graph formats. The coordinators converted this information to first decision trees and identified open issues which were discussed with the participant (see Fig. 3). In the majority of cases the first input received was not complete and in many cases the first input was logically inconsistent (with internal contradictions or several options without preference).

This process was repeated until all inconsistencies and gaps were resolved in the submitted decision tree. These inconsistencies included conflicting multiple recommendations for same combination of parameters or combinations of parameters for which a recommendation was not explicitly stated. Even though it is not intuitive, it is of utmost importance that during the process the coordinators do not judge the content of the decision trees as this might cause the participant to adapt the treatment recommendation and represents thus a potential source for bias. Also, the coordinators should never assume what the respondents meant without explicitly stating it. Within these projects we observed multiple treatment recommendations that were clearly beyond or even contradictory to well-established evidence in our view.

### Criteria adaptation

Criteria used in decision trees are standardized.

In some instances, similar criteria may be named differently by the participants. It is therefore important that the coordinators are experienced in the context of the study to mediate a common criteria adaptation among the participants where it seems appropriate without introducing further bias. For example, in the project on recurrent glioblastoma [17] some treatments were not recommended to patients that were considered “old” or had a “poor performance status” (see Table 1). After clarification with the participants an adequate replacement term - “fit” or “unfit” - was found. By using merged criteria, a meaningful comparison of different recommendations was facilitated. Otherwise “poor performance status” and “old” would need to be treated as independent factors. From our experience, including items like performance status, age, fitness and comorbidity as separate parameters may lead to unnecessary complexity without adding any value to the results.

**Table 1:** Example of different cut-off values for performance status, as initially collected for the trial investigating multimodal management of recurrent glioblastoma [17]

Center	Performance status	Cut-off value
A	KPS	</> 50
B	KPS	</> 60
C	KPS	</> 70
D	KPS	</> 90
E	ECOG	≤1,>1
F	no specific scale	good/bad

Potentially a new criterion may be discovered after the initial collection. In the renal cell cancer projects, several participants mentioned the need for fast treatment, each phrasing this criterion differently. We created a criterion termed “Zugzwang” [16, 21] (to be forced to any action) which was confirmed to be representative of their previous criteria. All centers were provided the opportunity to incorporate this criterion. This enabled a cross-comparison despite initially very different wording.

In all involved projects, certain criteria were defined by individual participants that would not improve the overall analysis but would lead to an exponential rise in complexity and manageability. An example for such a criterion was pulmonary fibrosis, a rare medical condition but a specific contraindication to a specific drug. For all finalized projects the coordinators defined an arbitrary threshold of at least e.g. three centers or guidelines mentioning a criterion to be included in the analysis. This threshold should be defined by practicability. If any criteria are excluded this needs to be well documented and specifically stated.

When a criterion applies to the overall setting, such as the patients’ informed consent to a treatment, this element should not be included in the tree analysis but extracted and discussed in the methodology or discussion of a manuscript.

### Problem re-evaluation

The initial research question is re-evaluated in light of collected decision trees.

After refinement of the decision trees and simplification and unification of decision criteria, the current result needs to be re-evaluated in respect to the initial problem definition. The initial problem definition was defined at a point where the decision criteria as well as recommendations of the participants were not known. In certain situations, a re-definition through specification of the initial problem may solve potential discrepancies. For example, some individuals may propose to include local treatments as alternatives to systemic treatment, which was not initially considered by the coordinators. If this is beyond the scope of interest or results in unmanageable complexity, specifying the problem as systemic treatment excluding local options may be a solution. Another example may be to re-define the problem by adding “in an otherwise healthy patient” to the definition of the clinical scenario when several, but rare, comorbidities are excluded as criteria.

This step is equally important in consensus analysis of guidelines. If, for example, several panels recommend multiple therapies for a specific situation and list them in a hierarchical manner, this may lead to unmanageably complex consensus decision trees. In such cases defining the “main recommended treatment” or “any recommended treatment” might bridge this gap.

### Results distribution

All used decision criteria are shared and participants are provided the option to incorporate these in their decision trees.

The aim of this step is to distribute all collected decision criteria – but not all recommendations - to each participant as well as their own recommendations in decision tree format for comparison. Despite due diligence on behalf of the participating centers, attention to unconsidered aspects may be drawn upon by the input of the other participants. For example, when active treatment was discussed, individual centers provided information on when to specifically not perform active cancer treatment and instead provide best supportive care. If these new aspects are included, all participants should have the opportunity of re-addressing these issues (see refinement Fig. 1).

### Tree finalisation

All decision trees are finalized at a specific date.

As patterns of algorithms studies can be performed in areas where controversy is expected, this may also be an area where strategies are constantly evolving, e.g. due to publications of trial results or due to updates of guidelines. The date of tree finalisation should be recorded to ensure correct versioning and context.

### Analysis

The decision trees are analysed for consensus and discrepancies.

**Fig. 4:**
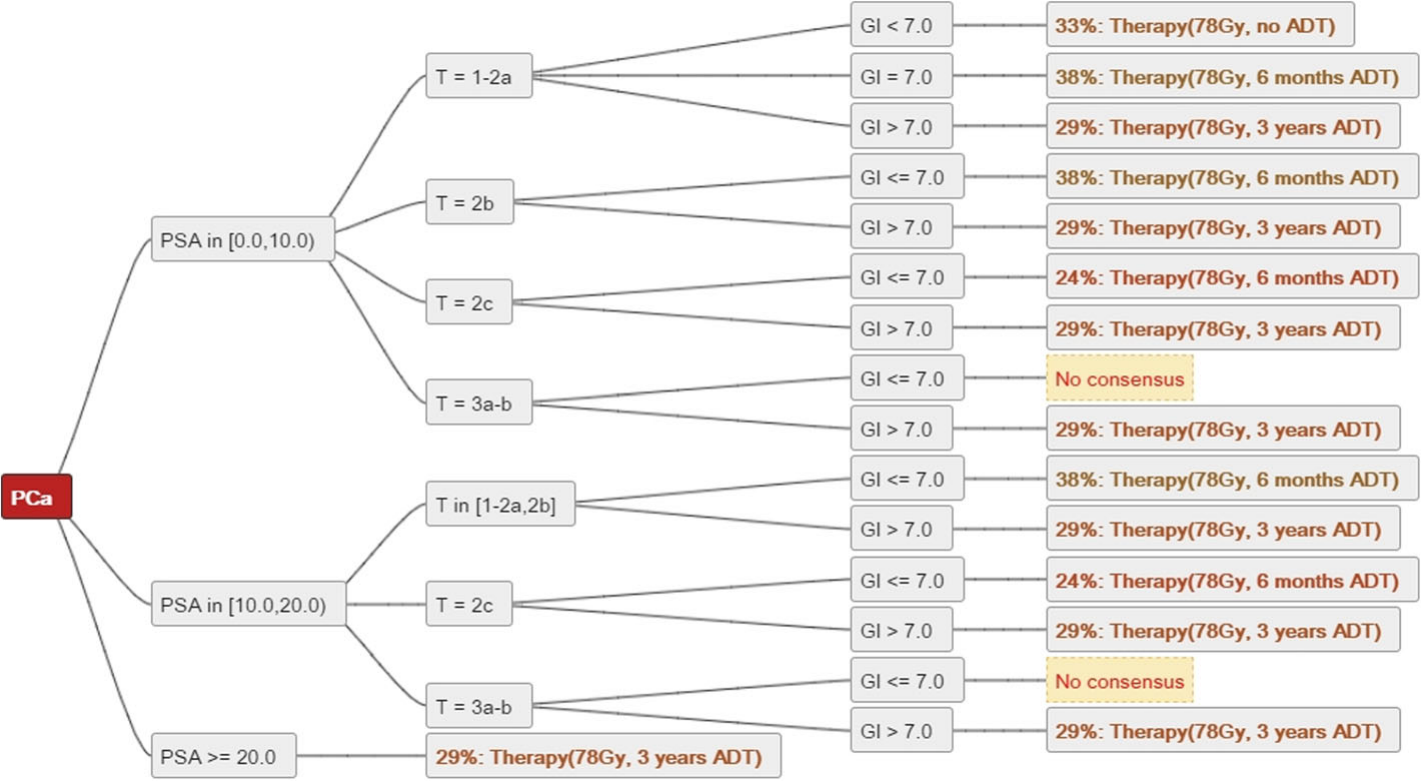
A mode decision tree summarising and quantifying the radiotherapy recommendations of 24 Swiss centers for localised prostate cancer [15]. Text below the figure: The figure shows very low consensus for radiotherapy dose and duration of androgen deprivation therapy based on PSA value, T stage and Gleason score in prostate cancer. PSA = Prostate-specific antigen level (μg/L). Gl = Gleason. ADT = Androgen deprivation therapy

The analysis of decision trees based on objective consensus can identify areas of controversy as well as consensus. In the provided example (Fig. 4) from the trial investigating the dose of radiotherapy and the length of androgen deprivation therapy no single combination of dose and length reached a majority, in several instances not even a single dominant combination was identified.

**Fig. 5:**
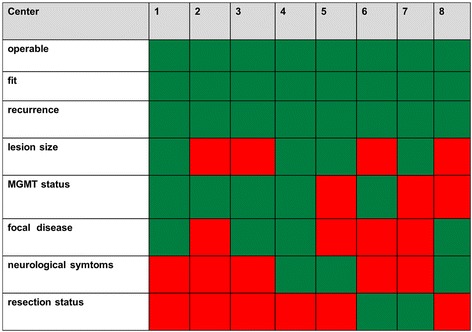
Profile of criteria being used in decision making for recurrent glioblastoma, adapted from Hundsberger et al. [17]. Text below the figure: Criteria used by a anonymised centers in decision making displayed by green squares, others in red. MGMT = O6-methylguanin-DNA-methyltransferase promotor methylation status

**Fig. 6:**
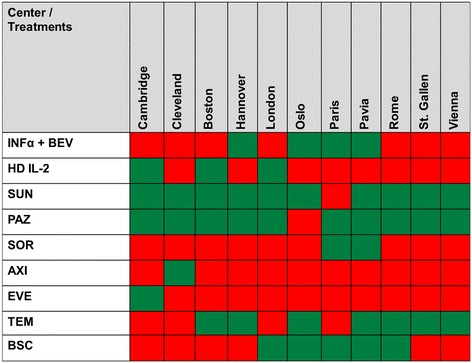
Treatment profile, the figures indicates the various treatments being offered in the context of first line metastatic renal cell cancer, adapted from Rothermundt et al. [21]. Text below the figure: Recommended treatment option from a specific center are displayed by green squares, others in red. The participating centres were named with their permission. INFα = interferon alpha; BEV = bevacizumab; SUN = sunitinib; PAZ = pazopanib; SOR = sorafenib; AXI = axitinib; EVE = everolimus; TEM = temserolimus; BSC = best supportive care

Besides recommendations the criteria implemented can be compared (Fig. 5), also the portfolio of treatments being offered by the participants may vary considerably and can be visualised (Fig. 6).

Apart from descriptive statistics (e.g. congruency rate in percentage for any decision), a clear definition of “consensus” is needed, such as the most common (mode) recommendation or a recommendation supported by more than 50% of the participating centers.

## Discussion

Evidence-based medicine is considered the gold standard for medical decision-making, but there are frequent situations where data are limited [3]. Consequently, many clinical decisions are based on consensus recommendations in the lack of high-level evidence [6, 22]. The objective consensus methodology as a means for consensus analysis, can automatically compare multiple treatment algorithms in the form of decision trees [14, 22].

There are several advantages of defining and analysing clinical decision trees in this manner.

First, the objective consensus methodology does not require a specific initial input format from the participating centres. A comprehensive questionnaire could facilitate the generation and individual decision trees; this is however impractical due to the exponential combination of implemented parameters. Also pre-defining criteria and their cut-off values might supress criteria used in practice.

The externalization of intrinsic knowledge and every-day know-how into the form of decision trees may cause problems [23]. It may therefore be beneficial to ask only for a short free-text version of treatment recommendations which can be then converted into a decision tree by the investigators and sent back for approval to the individual participant [14]. This approach has the advantage that the participants may not be biased in their treatment recommendations by previously circulating examples of decision trees.

By this means, a wide range of answers to potential parameter combinations can be covered.

Additionally, the analysis of treatment recommendation can be performed anonymously which may prevent a disproportionate influence of individuals on the process of consensus finding.

The objective consensus method provides a tool for consensus analysis where the effort per party remains constant potentially allowing an increased number of participants, which is in contrast to many traditional consensus finding methods [22].

The nominal group technique, which is also referred as expert panel, consists of a generation and collection of ideas concerning a specific topic with consequent discussion and ranking which is predominantly performed in a person-to-person meeting [22, 24]. Although it allows a high level of interaction and discussion between the participants, there are several drawbacks: the number of participants may be very limited for practicability reasons and therefore more biased than a consensus analysis in a larger collective. Additionally, the lack of anonymity may promote the influence of specific individuals and deter participants to express non-conformal opinions.

Another commonly applied methodology for consensus finding is the Delphi technique which provides a form of highly structured group interaction by completion of questionnaires in several rounds which is performed anonymously [24]. First, several statements on specific medical issues are provided by invited experts or based on a literature review and summarized in a questionnaire. Consequently, the participants rank their agreement with each statement (e.g. on a scale from 0 to 9). In a second round, the participants receive a summary of the collected rankings and can adapt their score in view of the responses of the other participants. The step of re-ranking can be performed several times until a high level of consensus is achieved [25]. The Delphi process shares with the objective consensus methodology the possibility for anonymous consensus analysis with a high number of participants. However, the objective consensus methodology deliberately avoids the disclosure of the group’s result to each participant as this may bias the original opinion and particularly affect participants with outlier positions. Additionally, the Delphi process can only evaluate the extent of agreement to predefined and well circumscribed statements, but it cannot adequately compare complex treatment algorithms (specifically the interaction of decision criteria). For instance, in a consensus analysis on recurrent glioblastoma, the Delphi progress could evaluate the level of agreement with a statement such as “re-irradiation is a treatment option for recurrent glioblastoma”. In contrast, the objective consensus methodology gives each participant the opportunity to use (or omit) individual factors and their combinations when to consider re-irradiation such as tumour size, location, previous therapy or performance status [17].

In daily routine, decision-making in controversial cases is often supported by clinical guidelines. However, interpretations of the available evidence can diverge in specific points leading to different guideline recommendations depending on the expert panel releasing them. Using consensus analysis, controversial areas can be detected where further research is needed [24]. Finally, the comparison of individual decision trees can equally present disagreement and consensus and may therefore depict controversial issues more comprehensively than traditional consensus finding methods which may remain inconclusive [14].

There are several limitations to this method. When compared to registry-based patterns of care trials, the numbers of specific situations are not collected and whether patients are treated as defined in algorithms also remains unproven.

Furthermore, treatment conditions of daily routine, which have not been addressed in clinical trials, can be addressed and agreed upon using the objective consensus method. Widespread clinical expertise from different physicians can be condensed in practical recommendations even though evidence is not available.

As mentioned in the section on problem definition, it is recommended to define a concise question when the objective consensus methodology is used: with too many parameters or situations, the method still works, but visualization and use of the results becomes more difficult, if not prohibitive. Therefore, the methodology is not feasible for very complex treatment decisions with consideration of multiple factors.

It should also be pointed out, that this method enables identification of consensus recommendations if this consensus is present. Identification of consensus is not its validation, as the majority opinion is not inherently correct.

Decision-tree based analyses provide a valuable means to summarise and quantify consensus for a given medical situation. The usage of criteria in decision making as well as the frequency of established treatments should be considered when considering feasibility and acceptance of clinical trials. Decision trees can serve to identify trends in interactions between criteria and recommendations in the clinical setting (e.g. via multiple correspondence analysis). By associating treatment, recommendations with costs and applying distributions to decision criteria health economic analyses become possible, including modelling impact of changes in parameter distribution (e.g. ageing population).

## Conclusion

In conclusion, the collection of decision trees and application of the objective consensus methodology requires several consecutive steps from problem definition over data collection and conversion to the final analysis, which should be carefully planned. Several published projects using this methodology demonstrate its feasibility. It is able to identify areas of consensus and heterogeneity/uncertainty, which can in turn serve as clinical research questions. Additionally, due to its simplicity it can also provide important information for health care authorities and health insurances to address reimbursement issues in areas where no evidence is available, such as rare diseases or rare clinical conditions.

## Abbreviations

ADT: Androgen deprivation therapy
AXI: Axitinib
BEV: Bevacizumab
BSC: Best supportive care
EVE: Everolimus
GEM: Gemcitabine
Gl: Gleason score
HD IL-2: High-dose interleukin 2
HI: Hepatic insufficiency
HIFU: High intensity focused ultrasound
MGMT: O6-methylguanin-DNA-methyltransferase promotor methylation status
PAZ: Pazopanib
PC: Pancreatic cancer
PS: Performance status
PSA: Prostate-specific antigen level
SOR: Sorafenib
SUN: Sunitinib
TEM: Temserolimus
ZZ: Zugzwang
